# Development of an Analytical Procedure for the Quantification of Artemisinin in Encapsulated Formulations

**DOI:** 10.3390/foods14244349

**Published:** 2025-12-17

**Authors:** Ana Šijanec, Matjaž Grčman, Matevž Pompe, Drago Kočar

**Affiliations:** Faculty of Chemistry and Chemical Technology, University of Ljubljana, Večna pot 113, SI-1000 Ljubljana, Slovenia

**Keywords:** artemisinin, encapsulation, food supplements, stability, HPLC-UV/Vis, quality control, sample preparation

## Abstract

Encapsulated formulations have emerged as a promising tool for increasing nutrient absorption in the food supplement and cosmetic industries. Although the theoretical amplification factors for improving the bioavailability of encapsulated formulations are very high for poorly soluble active compounds, it has long been known that encapsulation can also enhance the absorption of water-soluble ingredients. These findings have led to the development of new technologies for encapsulating nutrients for use in the food industry. However, accurate quantification of nutrients in encapsulated formulations in the food supplement industry remains a challenge. This study presents the development and validation of novel analytical procedures for determining artemisinin in various food supplement formulations. Three formulations were prepared using different emulsifying procedures for artemisinin encapsulation. High-performance liquid chromatography with UV/Vis detection (HPLC-UV/Vis) was used for analysis. Separation was performed using a Waters ACQUITY Premier BEH C18 column. Specialized sample preparation procedures were designed to efficiently disrupt encapsulation and extract artemisinin for precise quantification. Three different sample preparation procedures were required to accurately determine the artemisinin content in the tested formulations. All methods were validated. The precision, linearity expressed as R^2^, LOD, and LOQ of the chromatographic method were 0.39%, 0.9995, 18 µg/mL, and 26 µg/mL, respectively. Recoveries of the sample preparation methods were above 94%. The developed procedures enable accurate determination of artemisinin in encapsulated formulations, ensuring product quality and safety. These findings suggest that, for quality control of encapsulated food products, specialized analytical procedures for individual formulations may need to be developed and validated.

## 1. Introduction

Recently, the use of encapsulated formulations for delivering poorly soluble active ingredients or those with low biological efficiency has attracted considerable attention in the pharmaceutical [1] and food industries [2,3,4]. These advanced delivery systems offer increased solubility, improved bioavailability, and controlled release of active ingredients [5,6]. However, to ensure the efficacy and safety of such products, a comprehensive analysis of the formulations is required, particularly for quality control purposes.

Encapsulated formulations (e.g., micelles, liposomes, nanoemulsions) are self-assembled structures formed by amphiphilic molecules in aqueous solutions [7]. These structures have attracted considerable attention as drug delivery carriers because they can improve the solubility, stability, and targeted delivery of poorly soluble active ingredients [8,9]. When administered, hydrophobic active ingredients are encapsulated within the hydrophobic domains of these formulations, shielding them from the surrounding aqueous environment and preventing aggregation and degradation [10]. This configuration allows encapsulated delivery systems to effectively solubilize and protect nutrients from the environment before absorption. In the case of injectable pharmaceutical formulations, micelles can ensure the stability of active ingredients during circulation, facilitating their transport to the target site. Moreover, they enable efficient penetration into biological barriers, such as cell membranes, enhancing drug delivery to specific tissues or cells [11]. The controlled release of the drug from encapsulated formulations at the target site further ensures optimal therapeutic efficacy [12]. Through these mechanisms, encapsulated delivery systems hold great promise for overcoming various drug delivery challenges and advancing personalized and targeted medicine [13,14].

While drug encapsulation has been used for several decades, encapsulation technologies in the food industry have only recently begun to gain traction. This approach has proven particularly valuable in the food supplement sector, enabling the protection and controlled release of nutrients that are otherwise prone to degradation by stomach acid, enzymes, heat, and oxidation [15]. Encapsulation forms protective carriers that shield sensitive nutrients from harsh environmental conditions, ensuring their stability and potency until they reach their intended absorption site. This technology is especially effective for enhancing the bioavailability of lipophilic compounds, which typically have low solubility and absorption rates [16].

High-performance liquid chromatography with UV/Vis detection (HPLC-UV/Vis) is widely used for analyzing pharmaceutical and food supplement formulations due to its sensitivity and selectivity in detecting analytes [17,18]. However, when working with encapsulated formulations, a sample preparation step is necessary to disrupt the encapsulation and enable the release and detection of analytes. The encapsulated structure is stabilized by polar–polar interactions between the surfactant’s polar head and the surrounding aqueous medium, as well as nonpolar–nonpolar interactions between the nonpolar surfactant tails. These interactions are weakened by adding a less polar solvent that is miscible with water, causing the encapsulated structure to disintegrate [19]. The literature reports several methods for quantifying substances in encapsulated formulations using HPLC without special sample preparation. Pucci et al. (2003) reported the quantification of progesterone in encapsulated systems using a simple extraction procedure from commercial formulations and experimental encapsulated systems [20]. The procedure involved dissolving the encapsulated formulation in a water:2-propanol (1:1, *v*/*v*) mixture and diluting with the mobile phase, which consisted of a 2-propanol–phosphate buffer mixture at pH 3 (1:1, *v*/*v*) [20]. Yusuf et al. reported the quantification of curcumin in polymeric micellar powder using HPLC [21]. Disruption of the encapsulated structure for accurate quantification of curcumin was achieved by dissolving micellar powder formulations in methanol, followed by sonication until a clear solution was obtained. Chin et al. [22] and Jangle et al. [23] used Triton X-100 to disrupt liposomal formulations stabilized by PEG and cholesterol, respectively. As a nonionic surfactant (detergent), Triton X-100 facilitates the complete release of the encapsulated nutrient. Arakkunakorn et al. reported the quantification of dimethylcurcumin and resveratrol in Pluronic-F127 nanomicelles [24]. They prepared nanomicelles, freeze-dried them into solid samples, and then dissolved them in acetonitrile for RP-HPLC analysis. However, our preliminary studies showed that for certain encapsulated formulations, more sophisticated and tailored sample preparation techniques are necessary to achieve accurate and reliable results.

As a case study, we selected artemisinin, a natural sesquiterpene lactone derived from the Chinese medicinal plant *Artemisia annua* L., commonly known as sweet wormwood or qinghao. Artemisinin is a potent antimalarial drug that has revolutionized malaria treatment [25]. Beyond its antimalarial properties, artemisinin has also shown immunosuppressive, anti-inflammatory, antitumor, and antiparasitic activities [26,27]. With its therapeutic potential expanding, the demand for innovative artemisinin formulations is growing substantially, particularly to address artemisinin’s poor solubility in water. The literature reports the solubility of artemisinin in water as 59 μg/mL at 25 °C [28]. Lapenna et al. reported an increase in artemisinin solubility when using encapsulated formulations [29]. Artemisinin is used as an active ingredient in food supplements at subtherapeutic doses. Usually, it is not used as a pure ingredient but as a natural extract of the plant *Artemisia annua* L. A commercially available example is the formulation ArtemiC Rescue (Swiss Pharmacan AG), which contains three plant extracts, namely Boswellia serrata, Curcuma longa, and *Artemisia annua* L. extract. The encapsulated nature of formulations such as ArtemiC Rescue poses a challenge for sample preparation, as dilution with organic solvent is not sufficient to break up encapsulated formulations for accurate quantification of artemisinin.

The aim of this study is to develop and validate an analytical procedure for quantifying artemisinin in three different encapsulated formulations. The procedure is based on HPLC-UV/Vis and includes the optimization of sample preparation methods to ensure efficient disruption of encapsulation and reliable extraction of the active compound. The study focuses on model encapsulated formulations of *Artemisia annua* L. extract containing artemisinin as the nutrient, prepared using different encapsulating agents representative of systems used in food supplements. The developed approach provides a foundation for establishing reliable quality control procedures for encapsulated formulations in the food supplement industry.

## 2. Materials and Methods

### 2.1. Chemicals

Toluene and hexane (both HPLC grade), ethanol (HPLC gradient grade), acetonitrile (LC-MS grade), and polysorbate 80 were purchased from J. T. Baker (Phillipsburg, NJ, USA). Ascorbic acid of p.a. quality and o-phosphoric acid (85%, HPLC) were obtained from Carlo Erba (Cornaredo, Italy). Zinc nitrate hexahydrate of p.a. quality was sourced from Fluka (Buchs, Switzerland), and ammonium thiocyanate (p.a. quality) from Chemlab (Zedelgem, Belgium). Deionized water was purified with a Milli-Q purification system from Millipore (Burlington, MA, USA) before use.

Artemisinin standard (>98.0% purity) and *Artemisia annua* L. extract were purchased from Sigma Aldrich (St. Louis, MO, USA) and Bionex (Antananarivo, Madagascar), respectively. Lecithin P45 and lecithin H100 were purchased from Lipoid (Ludwigshafen, Germany), polysorbate 80 from Merck (Darmstadt, Germany), glycerol from Stella Tech (San Pedro Garza Garcia, Mexico), MCT and medium-chain fatty acid from Healthife LCC (Xi’an, China), L-Arginine from Vitalundfitmit100 GmbH (Jüchen, Germany), and sodium ascorbate from Buxtrade GmbH (Buxtehude, Germany).

The food supplement ArtemiC Rescue was donated by Swiss Pharmacan AG (Frauenfeld, Switzerland).

### 2.2. Preparation of Encapsulated Formulations

For the preparation of encapsulated formulations, several emulsifiers commonly used in the food industry were selected based on an analysis of chosen commercial products.

Encapsulated *Artemisia annua* L. extract formulation 1 (EnExART1) was prepared by mixing *Artemisia annua* L. extract (0.6% *w*/*v*) with lecithin P45 and dissolving the mixture in methanol. The organic solvent was then removed by rotary evaporation. The resulting dry thin film was hydrated with Milli-Q water at 60 °C for 1 h, and the suspension was left overnight at 4 °C. In the final step, the suspension was mixed with a homogenizer (Heidolph SilentCrusher M, Schwabach, Germany) at 12,000 rpm for 30 min. The solution was cooled in an ice bath to prevent the temperature from exceeding 70 °C.

Encapsulated *Artemisia annua* L. extract formulation 2 (EnExART2) was prepared by first dissolving *Artemisia annua* L. extract (0.6% *w*/*v*), lecithin P45, lecithin H100, and MCT oil. The mixture was stirred overnight at 40 °C. Subsequently, Milli-Q water containing L-arginine (5.0% *w*/*v*) and medium-chain fatty acid (7% *w*/*v*) was added and mixed with a homogenizer at 12,000 rpm for 30 min. The solution was cooled in an ice bath to prevent the temperature from exceeding 70 °C. In the final step, sodium ascorbate and glycerol (30.0% *w*/*v*) were added to adjust the pH to 5.

Encapsulated *Artemisia annua* L. extract formulation 3 (EnExART3) was prepared by mixing polysorbate 80 (20% *w*/*v*) and *Artemisia annua* L. extract (0.6% *w*/*v*) at 40 °C until homogeneous. Milli-Q water was then added, and the mixture was homogenized to achieve a transparent solution (8000 rpm for 10 min).

Particle size was determined using dynamic light scattering. Measurements were performed with a NIBS High-Performance Particle Sizer (ALV GmbH, Tettnang, Germany) equipped with a 3 mW He-Ne laser at 633 nm wavelength and a scattering angle of 173°. Samples were prepared by diluting the original formulations with Milli-Q water (1:100, *v*/*v*). The average particle sizes determined were 170 nm ± 5 nm for EnExART1, 38.1 nm ± 0.2 nm for EnExART2, and 28.0 nm ± 0.2 nm for EnExART3. In all three cases, monodispersed distributions were observed.

### 2.3. Preparation of Standards and Sample Solutions

The stock standard solution was prepared by dissolving artemisinin standard in ethanol at a concentration of 5 mg/mL, and then further diluted with ethanol to obtain working standard concentrations of 1.25, 1.00, 0.75, 0.50, 0.25, and 0.10 mg/mL. The stock standard solution was stable for at least 7 days, while working standards were prepared daily.

Solutions for stability testing were prepared by dissolving artemisinin standard in a solvent mixture of ethanol and water (2:3, *v*/*v*) at a concentration of 1 mg/mL, along with ascorbic acid in equimolar amounts.

The *Artemisia annua* L. extract solution was prepared by dissolving *Artemisia annua* L. extract in ethanol at a concentration of 3 mg/mL.

To quantify artemisinin in encapsulated formulations, several sample preparation techniques were tested.

Dilution with organic solvents: 1 mL of the encapsulated formulation was diluted tenfold with different organic solvents, including ethanol, acetone, acetonitrile, and isopropanol.

Extraction: 1 mL of the encapsulated formulation was extracted with 5 mL of toluene or hexane. After thorough mixing, 3 mL of the organic phase was collected and evaporated under a stream of nitrogen. The dry residue was then reconstituted with ethanol to a final volume of 1 mL.

Extraction with additional precipitation: 1 mL of encapsulated formulation was extracted with 150 mL of Milli-Q water and 50 mL of toluene or hexane. During extraction, 50 mL of precipitating solution was gradually added while shaking. The precipitating solution contained NH_4_SCN (19.0% *w*/*v*) and Zn(NO_3_)_2_ (6.0% *w*/*v*). After thorough mixing for 15 min, a white precipitate formed. Then, 3 mL of the organic phase was collected and evaporated under a stream of nitrogen. The dry residue was reconstituted with ethanol to a final volume of 1 mL. Extraction with additional precipitation was also used for sample preparation for the analysis of the food supplement ArtemiC Rescue.

### 2.4. HPLC Measurements

A Thermo Scientific Dionex UltiMate BIO-RS HPLC system, equipped with a binary pump (HPG-3400RS), an autosampler (WPS-3000 SplitLoop RS), a column thermostat (TCC-3000), and a diode array detector (DAD-3000RS), was used for chromatographic separation. All modules of the chromatographic system were purchased from Thermo Fisher Scientific Inc., Waltham, MA, USA. Chromeleon 7.2 (Thermo Fisher Scientific) software was used for data processing.

Analysis of samples was performed on a reverse-phase Waters ACQUITY Premier BEH C18 column with dimensions of 2.1 × 150 mm and a particle size of 1.7 µm. The flow rate was set to 0.2 mL/min, the injection volume to 1 µL, and the column temperature to 45 °C. UV detection was performed at a wavelength of 190 nm without using a reference wavelength. Sampling was done at 5 Hz. The mobile phase consisted of 0.1% phosphoric acid in Milli-Q water (A) and acetonitrile (B). The following gradient elution program was used to achieve baseline separation: 0–2 min, 50% B; 2–12 min, 50–95% B; 12–19 min, 95% B; 19–20 min, 95–50% B; and 20–35 min, 50% B.

### 2.5. Method Validation

The standard deviation (SD) was calculated as the square root of the sum of the square deviations of each measurement from the mean value, divided by the number of measurements minus one [30]:

$$SD=\sqrt{\frac{\sum_{i=1}^{n}(x_{i}-\bar{x}{)}^{2}}{n-1}}$$

The relative standard deviation (RSD) was calculated as the ratio of the standard deviation to the mean of replicate measurements, multiplied by 100 to express the result as a percentage [30]:

$$RSD(\%)=\frac{SD}{\bar{x}}\times 100$$

The limit of detection (LOD) and limit of quantitation (LOQ) were determined using the signal-to-noise (S/N) ratio method, following the ICH Q2 (R1) guidelines [31]. The LOD corresponded to an S/N ratio of 3, while the LOQ was defined at an S/N ratio of 10. The S/N ratio was obtained from the chromatogram by comparing the height of the analyte peak to the baseline noise near the retention time of artemisinin.

Recovery was calculated as the ratio of the experimentally determined amount to the theoretical amount, expressed as a percentage:

$$Recovery(\%)=\frac{\gamma_{found}}{\gamma_{added}}\times 100$$

Reproducibility was evaluated by performing three parallel quantifications for each sample. The mean value and relative standard deviation (RSD) from these triplicate analyses were used to assess the method’s precision. All data processing and statistical calculations were performed using Microsoft Excel (Microsoft Office 365, Redmond, WA, USA).

## 3. Results and Discussion

### 3.1. Optimization of Separation

**Figure 1:**
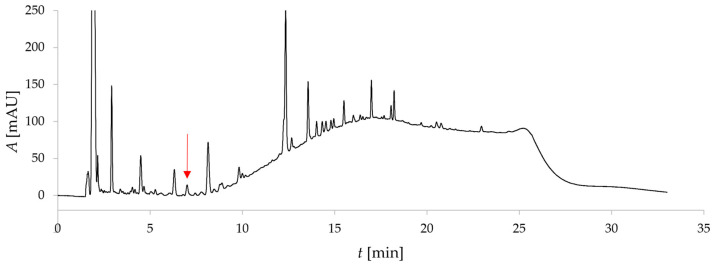
Chromatogram of the *Artemisia annua* L. extract. The artemisinin peak is indicated with a red arrow (*t*_r_ = 8.2 min).

The aim of this study was to develop an analytical method for quantifying artemisinin in various encapsulated formulations. To ensure reliable quantification of artemisinin, it was first necessary to develop and validate a chromatographic method capable of effectively separating artemisinin from other compounds in the samples. Since the encapsulated formulations contain the *Artemisia annua* L. extract as the source of artemisinin, the chromatographic separation was developed using non-encapsulated *Artemisia annua* L. extract as the reference sample. This approach ensured that artemisinin could be accurately distinguished from matrix interferences, allowing for its precise quantification in the analyzed samples. Several separation columns were tested using C18 and C8 stationary phases. The best selectivity and peak symmetry were obtained with the Waters ACQUITY Premier BEH C18 (2.1 × 150 mm) separation column with a particle size of 1.7 µm. Gradient elution was applied using water and acetonitrile as mobile phases A and B, respectively. The details of the chromatographic separation are described in Section 2. Figure 1 presents the chromatogram of *Artemisia annua* L. extract after optimization of the separation conditions.

After chromatographic separation was achieved, the method was validated to confirm its reliability for accurate quantification. The validation parameters obtained are presented in Table 1. The precision of the chromatographic method, reported as % RSD, was assessed by measuring repeatability with six replicate injections at a concentration of 1 mg/mL. The RSD was found to be 0.39%, indicating good precision of the analytical method. For linearity evaluation, a six-point calibration curve was constructed using the standard solutions described above in the 0.10–1.25 mg/mL concentration range. The calibration curve was linear within the tested range, with a correlation coefficient (R^2^) of 0.9995. LOD and LOQ values were calculated based on the signal-to-noise (S/N) ratio from the chromatogram of the sample solution to estimate these parameters under worst-case conditions. Noise was evaluated from baseline fluctuations before the artemisinin peak, where no visible elution of interfering compounds occurred. LOD and LOQ were determined as 6 and 10 times the S/N value, respectively. The corresponding values for LOD and LOQ were 18 μg/mL and 26 μg/mL. Additional hromatographic parameters for analysis of 3 mg/mL *Artemisia annua* L. extract are given in Table 2.

The accuracy evaluation was the final stage of method validation. It was performed on the *Artemisia annua* L. extract. The extract was spiked with artemisinin standard solution to achieve an approximate 50% increase in the chromatographic peak. Quantification was conducted with and without the spike in three replicates. The analysis showed that the artemisinin content in the *Artemisia annua* L. extract is 32.5%, with a standard deviation of 0.64%. This provides a basis for calculating the theoretical artemisinin concentration in the prepared encapsulating formulations. The accuracy, calculated as the ratio of the determined quantity of artemisinin added to the *Artemisia annua* L. extract versus the actual amount, was 98.8%, with a standard deviation of 1.0%. Based on these results, we can state that the developed procedure is accurate for quantifying artemisinin in the plant extract.

The validated chromatographic method was then applied to the commercially available food supplement ArtemiC Rescue. The sample was diluted tenfold with ethanol to ensure the breakdown of encapsulation. Unfortunately, the artemisinin content determined in the ArtemiC Rescue formulation using the described sample preparation procedure was close to zero.

Initially, it was unclear whether these results were due to degradation of artemisinin in the formulation or inaccuracy of the analytical procedure. In the first case, artemisinin may be degraded in the presence of other ingredients in the sample or due to technological parameters used during formulation production. Inaccuracy of the analytical procedure could result from incomplete release of artemisinin from the encapsulation.

The unexpectedly low recovery of artemisinin in the commercial formulation raised concerns about the suitability of applying general analytical validation procedures to encapsulated systems. This finding indicated that standard sample preparation procedures commonly used for non-encapsulated products may produce misleading results when applied to encapsulated formulations. To address this, three different formulations containing *Artemisia annua* L. extract (EnExART1, EnExART2, and EnExART3) were prepared, each based on a distinct surfactant system, and various sample preparation approaches were evaluated to achieve quantitative release and reliable quantification of the analyte.

Therefore, our further investigations addressed two suspected issues: the stability of artemisinin and its quantitative release from the formulation.

### 3.2. Stability Study of Artemisinin

The stability of artemisinin was tested under the production conditions of encapsulated formulations. During the encapsulation process, artemisinin is exposed to elevated temperatures, which could potentially accelerate degradation and result in lower concentrations of artemisinin in the formulations.

To achieve homogeneity in the encapsulated formulations, the preparation requires heating: EnExART1 was hydrated at 60 °C for 1 h, while EnExART2 and EnExART3 were stirred overnight at 40 °C. It was essential to evaluate whether artemisinin remains stable at these working temperatures, as any thermal degradation could affect its accurate quantification. In our study, we also included higher temperatures to determine whether they could be used in sample preparation for quantitative analysis without causing degradation of artemisinin.

For this purpose, a standard solution of artemisinin was stored at 25 °C (room temperature), 40 °C, 60 °C, and 80 °C, and its stability was monitored over time (1, 3, 5, 7, 9, and 24 h). The results showed that artemisinin is thermally stable at room temperature and at 40 °C over a period of 24 h. This confirms that the temperature used during the preparation of EnExART2 and EnExART3 does not lead to degradation of artemisinin. At 60 °C, artemisinin remained stable during the first seven hours of exposure, which corresponds to the conditions used for the preparation of EnExART1. However, exposure at 60 °C for 24 h caused a 6.5% degradation. At 80 °C, degradation occurred much faster, with 10.5% of artemisinin degrading within the first hour of exposure. These findings indicate that artemisinin can withstand short-term heating up to 60 °C, but prolonged exposure or higher temperatures are not suitable for processing.

One of the additives frequently found in dietary supplements is vitamin C, an antioxidant that can potentially react with artemisinin, an oxidant. In encapsulated formulations, these two compounds are separated by the carrier structure, but disruption of the encapsulation required for further analysis brings them into direct contact. Therefore, it was necessary to evaluate whether a redox reaction could occur between vitamin C (antioxidant) and artemisinin (oxidant) upon disruption of encapsulation, as such a reaction could interfere with the accurate quantification of artemisinin in more complex commercial products.

Initial studies conducted at room temperature (25 °C) revealed that when vitamin C was added to an artemisinin solution, no degradation occurred within the first 48 h. This result indicated that disrupting encapsulation for the purpose of accurately quantifying artemisinin in encapsulated formulations would not immediately trigger a redox reaction when both compounds are in direct contact. However, after 6 days, the degradation of artemisinin in the presence of vitamin C led to a 7% decline in artemisinin. Although this degradation is significant, the finding suggests that vitamin C alone does not induce an immediate redox reaction with artemisinin during the analytical procedure; only prolonged exposure may lead to its degradation.

The stability tests confirmed that artemisinin remains stable under the temperature conditions used for the preparation of encapsulated formulations of *Artemisia annua* L. extract, as well as in the presence of vitamin C as an additive in commercially available ArtemiC Rescue, eliminating degradation as a potential cause for reduced recoveries. This finding was crucial, as it ensured that any lower yield observed in subsequent analyses would result from incomplete release of artemisinin from the encapsulated formulations, rather than from compound degradation. With these findings, the next step was to develop sample preparation procedures for EnExART1, EnExART2, and EnExART3, aimed at achieving quantitative release and reliable quantification of the active compound.

### 3.3. Development of an Analytical Procedure for the Quantification of Artemisinin in Encapsulated Formulation

Three different encapsulated formulations of *Artemisia annua* L. extract (EnExART1, EnExART2, and EnExART3), each using a distinct surfactant system, were investigated to develop efficient sample preparation procedures. The aim was to identify conditions that would enable quantitative release of artemisinin from the encapsulated structures and thus ensure accurate quantification by HPLC-UV/Vis. For each formulation, several sample preparation strategies were systematically tested and compared. The recovery of released analyte from the encapsulated structures was calculated as the ratio of artemisinin quantified in the sample to the theoretical amount added during formulation preparation. EnExART1 was based on soy lecithin P45; EnExART2 combined soy lecithin P45 and sunflower lecithin H100 with additional stabilizers (L-arginine and medium-chain fatty acid); EnExART3 used the synthetic surfactant polysorbate 80. Since no validated method for quantifying artemisinin in encapsulated formulations has been reported, we began with a simple dilution using an organic solvent to disrupt the encapsulation structure and perform HPLC analysis of the released analyte.

The encapsulated formulations were first diluted tenfold with different organic solvents, with ethanol providing the most promising results, as shown in Table 3. However, the results varied considerably between formulations. For EnExART1, the recovery of artemisinin reached 86.0%, while EnExART2 showed a slightly higher recovery of 94.2%, indicating that, in this case, ethanol was sufficient to achieve quantitative release. In contrast, EnExART3 yielded only traces of artemisinin, with the signal below the limit of detection. These findings suggest that ethanol dilution can be effective for certain surfactant systems, but changing the polarity of the solvent alone was not sufficient to disrupt every encapsulation structure.

To further improve release efficiency, liquid-liquid extraction was tested to quantitatively remove artemisinin from the aqueous system. We selected toluene as the extraction solvent because it is immiscible with water and has a high solubility for artemisinin (90.7 g/L [32]). After extraction, the organic phase was removed by evaporation, and the sample was redissolved in ethanol. This approach significantly enhanced the recovery for EnExART1, achieving 99.3% recovery. Recovery for EnExART2 was 81.6%, while EnExART3 again showed no detectable levels of artemisinin. This contrast in release performance can be attributed to the inherent physicochemical differences between natural lecithin and the synthetic polysorbate 80 system. Natural lecithin, a complex mixture of phospholipids and other lipid components, tends to form heterogeneous encapsulated structures, which often exhibit lower physical stability [33]. In contrast, polysorbate 80 is a chemically uniform synthetic surfactant known to form highly stable nanoemulsions and micellar structures [34]. This difference explains why simple dilution or extraction was insufficient for complete artemisinin release in EnExART3.

While in EnExART1 and EnExART2, artemisinin was successfully released from encapsulated structures using ethanol dilution and toluene extraction, EnExART3 proved to be more challenging. It appeared that artemisinin was strongly stabilized within the encapsulation structure, and successful extraction required removal of the emulsifier from the sample. One possibility was to precipitate polysorbate 80 based on the method described by Jäpelt and Christensen [35]. Polysorbate 80 was precipitated by adding a solution of Zn(NO_3_)_2_ and NH_4_SCN to the diluted EnExART3 in ethanol. Zn(NO_3_)_2_ was added to form a complex with polysorbate, and NH_4_SCN was used for precipitation. However, even after this step, no artemisinin peak was observed on the chromatogram, although the formation of a precipitate was detected visually.

Based on the results, it was clear that introducing an additional distribution process to quantitatively remove artemisinin from the observed system was necessary. We selected toluene as the extraction solvent. The artemisinin response for EnExART3 was finally obtained (with a recovery of 97.7%) after precipitating polysorbate 80 from the aqueous sample using NH_4_SCN and Zn(NO_3_)_2_ solution, while simultaneously extracting with toluene. The extraction was performed in a separatory funnel with constant shaking for 10 min. Due to the significantly higher solubility of artemisinin in the organic phase, efficient extraction was achieved. After extraction, the organic phase was removed by evaporation, and the sample was redissolved in ethanol.

To confirm that the precipitation step specifically targeted polysorbate 80 and was not required for the lecithin-based formulation, the same procedure was applied to EnExART1 and EnExART2. According to [35], the precipitate forms only between polysorbate 80, zinc, and thiocyanate. We confirmed that in both cases, where the surfactant was lecithin-based, no precipitation was observed, and the recoveries remained the same as those obtained with toluene extraction alone.

It was not possible to present chromatograms of intact vs. disrupted formulations, since the organic mobile phase used in HPLC destabilizes encapsulated structures during chromatographic separations. For this reason, the efficiency of each sample preparation method was evaluated exclusively based on the recovered amount of artemisinin after disruption.

For each encapsulated formulation, the most suitable sample preparation procedure was identified. The highest recovery for EnExART1, EnExART2, and EnExART3 was achieved by toluene extraction, ethanol dilution, and a combined precipitation–extraction approach, respectively. Each optimized procedure was performed in triplicate. Accuracy was calculated as the ratio of found concentration to the added concentration of artemisinin. The accuracies were 99.3% with an RSD of 1.2% for EnExART1, 94.2% with an RSD of 1.3% for EnExART2, and 97.7% with an RSD of 0.3% for EnExART3. It should be noted that in-house reference standard formulations were prepared, and the reported recoveries represent the best estimation of the accuracy of the measurements.

Results show that although the same active compound is used, different surfactant systems require specific sample preparation to achieve quantitative analyte release. Comparison of analyte release across EnExART1, EnExART2, and EnExART3 indicated that each formulation required a tailored sample preparation procedure to quantify artemisinin. Interactions between the nutrient and the encapsulating excipients, therefore, appear to contribute significantly to the robustness of the encapsulation.

After establishing optimized sample preparation procedures for our three encapsulated formulations of *Artemisia annua* L. extract, the next step was to evaluate whether these methods could also be applied to a commercially available product. For this purpose, the food supplement ArtemiC Rescue was selected, as it represents a relevant case study due to its complex composition. In ArtemiC Rescue, *Artemisia annua* L. extract is encapsulated along with Boswellia serrata and Curcuma longa, with polysorbate 80 acting as an emulsifier, similar to our formulation EnExART3. Additionally, the formulation contains vitamin C. Our stability test confirmed that vitamin C does not induce degradation of artemisinin within the analytical time frame, making the developed procedure suitable for the analysis of ArtemiC Rescue.

### 3.4. Quantification of Artemisinin in Commercially Available Food Supplement ArtemiC Rescue

To ensure reliable quantification of artemisinin in the food supplement ArtemiC Rescue, it was essential to validate the developed protocol established using the model formulation EnExART3. Because this system required a combined precipitation–extraction procedure to achieve complete release of the analyte, validation was necessary to confirm the reliability of the method.

First, the repeatability of the extraction procedure was assessed to evaluate the method’s consistency. An encapsulated artemisinin standard (using polysorbate 80 as an emulsifier) at a concentration of 1.67 mg/mL, corresponding to a final concentration of 0.1 mg/mL after extraction and sample preparation, was subjected to the full protocol six times. The resulting RSD of 1.4% confirmed good repeatability and robustness of the method. For linearity evaluation, encapsulated artemisinin standard solutions were prepared at concentrations of 0.83, 1.67, 2.50, 3.33, and 4.17 mg/mL, corresponding to final concentrations of 0.05, 0.10, 0.15, 0.20, and 0.25 mg/mL after extraction. The calibration curve was linear over the tested concentration range, with a correlation factor (R^2^) of 0.9995.

As a final step, accuracy was assessed. An encapsulated mixture of *Artemisia annua* L. extract and a known amount of artemisinin standard was prepared to achieve approximately a 50% increase in peak area. This mixture was then subjected to the extraction-precipitation procedure. The procedure was performed in triplicate, and the accuracy, calculated as the ratio of determined to added artemisinin, was 97.0%, with an RSD of 1.3%.

The present study focused primarily on establishing the feasibility and accuracy of extraction procedures; robustness and intermediate precision were beyond the scope of the current study but represent the next step for full regulatory validation.

Once the method was validated, we determined the extraction yield of artemisinin from the food supplement ArtemiC Rescue. In this case, the theoretical content of artemisinin was not defined by our formulation process but was taken from the manufacturer’s certificate. The extraction, followed by precipitation with NH_4_SCN and Zn(NO_3_)_2_, was repeated three times. The extraction resulted in a recovery of 93.5% of the declared value, with an RSD of 1.6%. The investigation showed that incorrect sample preparation, which does not release the studied ingredients quantitatively, not only lowers the obtained results but can also incorrectly indicate the absence of the ingredient in the prepared formulation. The strong stabilization of the encapsulated formulation likely results from strong lipophilic interactions between the nonpolar ingredient and the nonpolar tails of the emulsifier. These interactions will occur only with specific ingredient–emulsifier combinations. Such interactions are difficult to anticipate in advance; therefore, thorough accuracy testing is always necessary when analyzing any encapsulated formulation.

## 4. Conclusions

The aim of this study was to develop and validate a reliable analytical procedure for the assay of artemisinin in encapsulated food supplement samples. Chromatographic separation was optimized using *Artemisia annua* L. extract as a reference, achieving high selectivity and precision with a Waters ACQUITY Premier BEH C18 column. Validation of the developed method showed excellent selectivity, linearity (R^2^ = 0.9995), precision (RSD = 0.39%), and accuracy (98.8% recovery), ensuring reliable quantification of artemisinin. However, when the method was applied to encapsulated formulations, it did not provide accurate results. Further testing ruled out production conditions as a source of artemisinin degradation and indicated that incomplete release of active compounds from the encapsulated formulation was the most probable cause of incorrect assay results.

To study this issue, three different encapsulations of artemisinin were tested to thoroughly investigate the problem. Each formulation was evaluated using various sample preparation procedures, each designed to disrupt encapsulation and quantitatively extract the analyte into the solution analyzed by the HPLC procedure. It was found that no single procedure is effective for all encapsulated formulations. This result was expected, as very different types of emulsifiers were used. Validation of the developed analytical methods was performed using optimized sample preparation procedures. The developed method was linear, precise, and produced accurate results. Each optimized extraction procedure achieved at least 94.2% accuracy.

This study provides reliable methods for the assay analysis of artemisinin in encapsulated food supplement formulations, ensuring reproducibility and accuracy of the results. However, the study also highlights the general problem of quality control for encapsulated formulations. The results show that, in some cases, tailored analytical procedures are required for each encapsulated formulation, making generalization or standardization impossible. To our knowledge, this is the first study to raise this issue. Strong interactions between the ingredient and the nonpolar tail of the emulsifier can be difficult to predict in advance; therefore, thorough accuracy testing is always necessary when analyzing any encapsulated formulation. The findings underscore the need for careful design of quality control procedures for determining nutrient content in novel encapsulated formulations, as older analytical procedures, although validated for regular food products, can yield incorrect results.

Given that sample preparation steps rely on readily scalable liquid–liquid unit operations, the workflow holds promise for transfer to pilot- or production-level quality control, pending future robustness testing. Further development is possible with a more detailed experimental design; however, the aim of the study was to demonstrate that different encapsulated formulations require different quality control procedures. The developed procedures are not necessarily optimal, as the focus of the study was not the quantification of artemisinin, but rather to present the necessary concept of quality control for encapsulated formulations.

## Figures and Tables

**Table 1:** Validation parameters of the chromatographic method.

Injection repeatability	0.39%
Linearity	R^2^ = 0.9995
LOD	18 μg/mL
LOQ	26 μg/mL
Accuracy (mean ± SD)	98% ± 1.0%

**Table 2:** Chromatographic parameters for analysis of 3 mg/mL *Artemisia annua* L. extract.

Retention time	8.20 min
Peak width	0.16
Tailing factor	1.18
Peak resolution	2.27
Theoretical plate value	14,459
γ_artemisinin_	(0.98 ± 0.02) mg/mL
Peak area	29.3712 mAU × min

**Table 3:** Recoveries of released artemisinin from different encapsulated formulations (EnExART1, EnExART2, and EnExART3) obtained with different sample preparation methods.

Sample Preparation Method	Recovery [%]		
	EnExART1	EnExART2	EnExART3
Ethanol dilution	86.0	**94.2 ± 1.2 ** ^†^	n.d. *
Acetone dilution	61.1	57.9	n.d. *
Isopropanol dilution	76.1	55.1	n.d. *
Acetonitrile dilution	61.7	31.8	n.d. *
Toluene extraction	**99.3 ± 1.2 ** ^†^	81.6	n.d. *
Toluene extraction + Zn(NO_3_)_2_ + NH_4_SCN	98.7	82.1	**97.7 ± 0.3 ** ^†^

* below the limit of detection. ^†^ values represent mean ± SD (*n* = 3). Numbers in bold represent the recoveries of the most suitable sample preparation procedure.

## Data Availability

The original contributions presented in the study are included in the article; further inquiries can be directed to the corresponding author.
